# Screen-printed flexible MRI receive coils

**DOI:** 10.1038/ncomms10839

**Published:** 2016-03-10

**Authors:** Joseph R. Corea, Anita M. Flynn, Balthazar Lechêne, Greig Scott, Galen D. Reed, Peter J. Shin, Michael Lustig, Ana C. Arias

**Affiliations:** 1 Department of Electrical Engineering and Computer Sciences, University of California, Berkeley, California 94720, USA; 2 Department of Electrical Engineering, Stanford University, Stanford, California 94305, USA; 3 Department of Bioengineering, University of California, San Francisco, California 94722, USA

## Abstract

Magnetic resonance imaging is an inherently signal-to-noise-starved technique that limits the spatial resolution, diagnostic image quality and results in typically long acquisition times that are prone to motion artefacts. This limitation is exacerbated when receive coils have poor fit due to lack of flexibility or need for padding for patient comfort. Here, we report a new approach that uses printing for fabricating receive coils. Our approach enables highly flexible, extremely lightweight conforming devices. We show that these devices exhibit similar to higher signal-to-noise ratio than conventional ones, in clinical scenarios when coils could be displaced more than 18 mm away from the body. In addition, we provide detailed material properties and components performance analysis. Prototype arrays are incorporated within infant blankets for *in vivo* studies. This work presents the first fully functional, printed coils for 1.5- and 3-T clinical scanners.

## Abstract (web summary)

Signal-to-noise ratio is one of the key factors that currently limit the diagnostic image quality and patient conditions of magnetic resonance imaging. Here, Corea *et al*. use fully printed flexible receive coils, conforming to patient bodies, to improve signals and patient comfort in clinical scanners.

## Graphical abstract

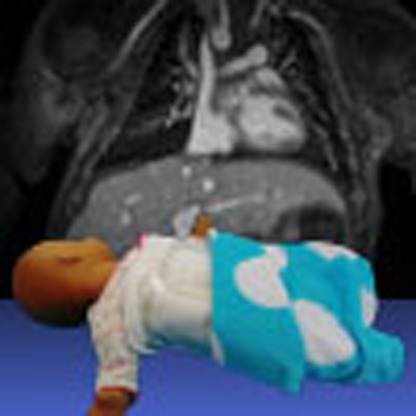

Magnetic resonance imaging (MRI) is a widely used non-invasive imaging modality that provides an unsurpassed variety of high-resolution soft-tissue contrast and functional information^1^,^2^. Unlike computed tomography, MRI scans do not expose patients to harmful ionizing radiation, and are considered safe^3^,^4^. Unfortunately MRI data acquisition is inherently slow and signal-to-noise ratio (SNR) starved. This limits the spatial resolution, diagnostic image quality and typically results in long acquisition times that are prone to motion artefacts. Recent advances in MRI such as parallel imaging^5^,^6^ and compressed sensing^7^ enable reduction in scan time by collecting less data and using advanced reconstruction techniques. However, these reductions are ultimately limited by the SNR obtained during the shorter scan. SNR can be increased by the use of contrast agents^8^,^9^ and higher-field scanners^10^, but better receive coils^11^,^12^ often provide more significant gains. A typical MRI paradigm consists of placing the patient in the large static field, giving rise to a net magnetic moment, using radio frequency (RF) pulses to excite the magnetization, which emits tiny amounts of RF energy at a characteristic resonant frequency proportional to the magnetic field, switching gradient magnetic fields to encode spatial information by manipulating the resonant frequency in three-dimensional space, and setting the spatial resolution and receiving a current signal from receive coils placed in close proximity to the body via Faraday induction throughout the duration of the encoding time. These received RF signals, which represent partial coded information, are amplified, digitized and stored. This procedure is repeated with different gradient waveforms until enough information is collected to form an image. It is the rate in which gradients switch that sets the amount of data collected each time. This rate is fundamentally limited by physiological constraints leading to relatively long exams that are uncomfortable, limit patient access, increase cost and more importantly, make the acquisitions susceptible to motion artefacts. The use of coil arrays provides additional SNR and scan acceleration by parallel imaging^5^,^6^, mitigating the above shortcomings.

Surface receive coil and arrays are typically built to acquire images with the highest possible SNR for a specific area of the body^13^. Currently, the manufacturing process for commercial coils relies on the use of high-quality electronic components such as porcelain capacitors, thick copper traces and low-loss substrates. The electrical elements of each coil are packaged with medical grade, fire resistant materials that contribute to the size and weight of a given array. Figure 1a shows typical coil arrays used for head and chest imaging on an adult. If the same chest array were used on a smaller person or a child, there would be large gaps between the coil elements and the body, squandering much of the SNR gained from high-quality components. This problem is aggravated in small children, often requiring general anaesthesia to restrict motion during the exam. Therefore, good coil fit to obtain high SNR is often critical in shortening scan time and reducing complications^14^,^15^. For example, Fig. 1b illustrates the importance of coil placement on SNR. It compares a cervical spine image obtained by a printed flexible coil (right) that fits perfectly against the neck to one obtained using a conventional surface coil (left) mimicking a worse case scanning condition, placing it on the patient table 8 cm away from the base of the neck. This imaging case, while extreme, clearly highlights the importance of coil placement during a scan, showing a large loss in SNR from poor coil placement. When a receive coil is placed close to the body, its sensitivity to tissue signal is markedly increased. At the same time, the coil is strongly affected by the conductivity of human tissue, which can be modelled as additional resistive losses. The latter effect increases with field strength (frequency) and coil size to the point that in most typical clinical imaging scenarios, the losses due to the sample dominate the intrinsic losses in the system (shown in Supplementary Fig. 1 using Supplementary Equations 1 and 2)^16^,^17^. This provides an opportunity in which novel solution processed electronic materials, which previously have been dismissed due to higher loss, can still perform adequately for receive coils without compromising image SNR. At the same time, these materials could provide significant added value of flexibility, lightness and mass manufacturing ability. In the past, several works have focused on adding flexibility and conformity to MRI receive arrays using a conductor sewn into fabric^18^, a mercury-based conductor^19^,^20^ and semi-flexible copper tape^21^. The advantage that printing has over previous techniques is the scalability and adaptability it possesses, qualities necessary to become a commonly used technology. Here, we report a powerful new approach that uses printing for the design and fabrication of MRI receive coils. Advances in electronic materials processed from solution have resulted in the demonstration of flexible electronic devices such as light emitting diodes, thin film transistors and photovoltaic devices^22^,^23^,^24^,^25^,^26^. Flexible electronics applications targeting the consumer electronics market are very exciting, but when devices are in contact with the human body advantages given by flexibility add considerable functionality^27^,^28^,^29^. Our method addresses the imaging limitations by enabling highly flexible, lightweight devices that conform to the human body, much like bespoke garments.

## Results

### Fabricating MRI coils using screen-printing on flexible substrates

**Figure 1:**
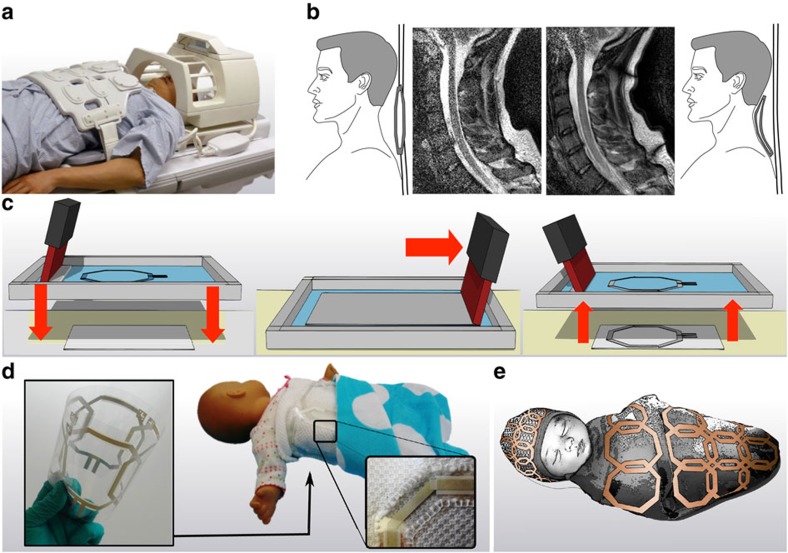
RF receive coil arrays proximity to body results in better image SNR. (**a**) Conventional MRI receive arrays on the chest and head of a patient. (**b**) Cervical spine images of volunteer showing low-SNR when using a coil placed 8 cm away from the spine (left) and high SNR when placed against the skin (right). (**c**) Schematic representation of fabrication process of flexible printed coils. The screen is patterned with emulsion (blue) and shows the coil design. Ink (grey) is transferred to the substrate (white) during the screen-printing process. (**d**) Photograph of a printed flexible four-channel coil array fabricated on plastic film and integrated into an infant blanket. The inset shows how a printed coil is stitched into the fabric. (**e**) Concept drawing of an infant swaddle and hat with an integrated printed receive coil array.

Printing can be tailored by using different inks, substrates and techniques enabling custom pattern design^22^. Inkjet printing has previously been used to deposit metal layers for a single-element receive coil designed for a high-frequency small animal system; however, inkjet printing does not scale well^30^. We use screen-printing because coils require thick, low resistance conductive traces over a large area (that is, body size) at a high throughput, something not easily achieved with inkjet printing. The coils demonstrated here are screen-printed onto lightweight flexible substrates as illustrated in Fig. 1c. In screen-printing, ink is forced through a pre-patterned mesh onto a substrate^23^ (Fig. 1c). To take advantage of flexibility and demonstrate feasibility, receive arrays were integrated with a baby blanket, as shown in Fig. 1d and used to scan volunteers. We envision that this technology could enable tightly fitting customized garments with integrated MRI coils, as illustrated in Fig. 1e. These could enable paediatric patients to receive shorter MRI exams with increased comfort and image quality.

### Printed MRI receive coils designed for 1.5 and 3.0 T scanners

**Figure 2:**
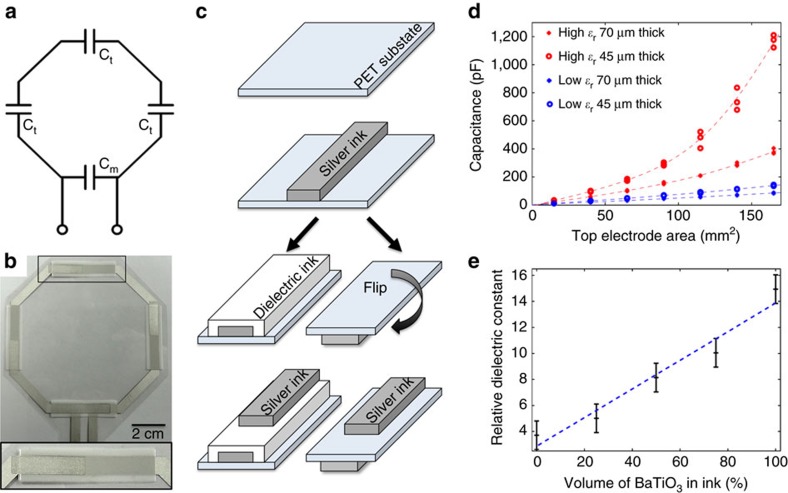
Fabrication method and characterization of printed receive coils. (**a**) Schematic of a printed coil showing tuning, C_t_, and matching, C_m_, capacitors. (**b**) Photograph of a printed coil. Inset highlights top-down view of printed capacitor. (**c**) Coil printing process flow showing two optional possible processes: printed dielectric or using the substrate as a dielectric. (**d**) Dependence of capacitance with top electrode area, dielectric thickness and ink composition. (**e**) Relative dielectric constant, measured at 127 MHz, as the volume of barium titanate in the ink is increased. High dielectric constant is achieved with barium titanate ink, while low dielectric constant is achieved with ultraviolet-curable ink. Error bars show standard deviation.

In the simplest sense, receive coils are formed by loops of wire integrated with capacitors. The resonant frequency of a conductive loop is determined by its inductance and capacitance, which both depend on the geometry and materials used. The size of the loop is typically predetermined, fixing the inductance. Therefore, tuning capacitors, C_t_, are added to the loop to tune the desired resonant frequency. To minimize cable losses a matching capacitor, C_m_, is added to match the input impedance to 50 Ω (refs 13, 31). The schematic representation of a typical MRI coil is shown in Fig. 2a. We use octagonal coils with a diameter of 8.7 cm (ref. 32), with a conductor width of 0.5 cm and four capacitors evenly spaced throughout the loop as shown in Fig. 2b. To fabricate the coils, we print the coils layer by layer from solution, illustrated in Fig. 2c. The first layer of conductive ink is printed onto a thin flexible substrate, typically polyethylene terephthalate, forming the metal loop of the coil. The coil is completed with matching and tuning capacitors by printing a dielectric layer and the top electrode metal layer. The metal ink is a conductive silver micro-flake solution (Creative Materials 118-09 A/B). The insulating dielectric ink is a mixture of barium titanate (Conductive Compounds BT-101) and a ultraviolet-curable resin-based ink (Creative Materials 116-20). When tuning a coil, it is desirable to control the capacitance to reach the Larmor frequencies used in MRI systems, ∼64 MHz (1.5 T) and 127 MHz (3.0 T). In our printed process, the area of the top electrode along with the thickness and composition of the dielectric layer can modify the capacitance. The dependence of capacitance with printed dielectric thickness (40 and 75 μm), composition of dielectric ink (*ε*_r_=15 and *ε*_r_=4) and top electrode area is summarized in Fig. 2d. We have found that relative dielectric constant increases linearly with the concentration of barium titanate in the ink, as shown in Fig. 2e. This experimental window allowed us to achieve capacitances ranging from 2 to 1,200 pF, matching the ranges needed for tuning coils to different frequencies with similar coil geometry. We concluded that the most effective strategy for coarse tuning the capacitance is the control of the composition of the dielectric ink while changing the area of top electrode provides the fine-tuning needed to reach the specific frequencies.

Currently, the materials for printed devices are constantly evolving and better-suited inks are becoming available for a wider variety of substrates. Fortunately, our printing process lends itself very well to rapid prototyping with these new materials without the need for completely redesigning the printing process. For example, it is possible to use the substrate as the dielectric for the printed capacitors without a significant change in coil design. To demonstrate this case, we created a coil with an improved conductive silver ink (Silver micro-flake, Dupont 5064H) printed on both sides of a low-loss substrate, polyether ether ketone (PEEK), forming capacitors wherever the layers overlap, shown in Fig. 2c.

### Signal-to-noise ratio of printed flexible coils

**Figure 3:**
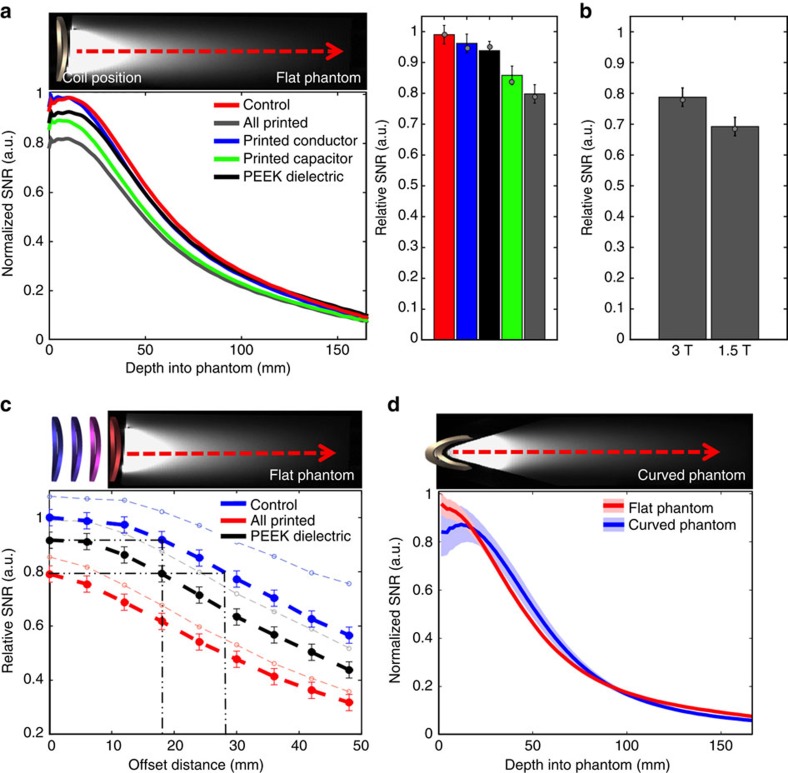
1.5- and 3-T scanner receive coil SNR characterization. (**a**) Normalized SNR versus depth into the phantom for coils fabricated with different permutations of printed components at 3 T, with schematic showing coil position 3 mm away from conductive fluid. Bar graph summarizes trends shown in relative SNR for each coil type. Dot on bar graph shows predicted SNR extracted from bench top quality factor measurements. (**b**) Relative measured (bars) and bench top-predicted (dots) image SNR of printed coils at 1.5 and 3 T. (**c**) Relative SNR for control and printed coils versus increasing coil offsets from the top surface of the phantom. Black dashed-dotted line highlights the position offset where the control coil shows equal SNR to the printed coils when the printed one has no offset from the top surface of the phantom. Light thin lines represent calculated best case performance when preamplifiers are added to the coil. Error bars show standard deviation. (**d**) Average normalized SNR profile for printed coils flexed around the surface of a curved saltwater phantom (blue) and placed on a flat phantom (red) at 3 T. Wide coloured bands indicate the s.d. across several coils.

The viability of using our printed flexible coils in a clinical setting was first evaluated by characterizing image SNR, using a NiCl_2_-doped saltwater (0.68 S m^−1^ at 3T) phantom as a model for human tissue. In our study, we fabricated five different types of coils as follows: an all-printed flexible coil; a copper coil in which printed capacitors replaced the conventional capacitors; a coil with printed silver conductors integrated with low-loss porcelain capacitors; a coil with improved printed silver conductors utilizing the low-loss PEEK substrate as the dielectric for capacitors; and a semi-flexible control coil composed of copper conductors and low-loss porcelain capacitors. All coils had the same geometry, and the control coil was not placed in any mechanical enclosure, allowing us to flex and measure SNR with all five types of coils. The SNR for each type of coil was calculated based on measured quality factor (Q) and by the method described in Hayes *et al*.^33^ The loaded Q values (measured in close proximity to the phantom) were 6.7 (fully printed), 7.6 (printed capacitors), 9.7 (printed conductors, discrete capacitors), 9.5 (PEEK dielectric, printed conductors), and 11.4 (non-printed). Unloaded Q values are shown in Supplementary Tables 1 and 2. A diagram illustrating the experimental imaging set up and relative SNR in cross-section through the phantom are shown in Fig. 3a. We found that at 3 T and close to the phantom, surface coils present 79±3%, 86±3%, 93±3% and 96±3% relative SNR corresponding to fully printed, printed capacitors, PEEK capacitors and printed conductor, respectively. SNR values are normalized with respect to the control coil, and the SNR predicted from bench tests is shown as a dot in the bar graph of Fig. 3a. The fully printed coils show slightly higher relative SNR when used at 3-T compared with the 1.5-T system, as shown in Fig. 3b. This difference is attributed to the larger role that coil loss plays at lower frequencies as described in Darrasse *et al*.^16^. The fully printed and control coils were placed at increasing offsets from the phantom and the SNR was measured in actual scanned images. As expected and shown in Fig. 3c (tabulated in Supplementary Tables 3–6), SNR decreases as the distance from the phantom increases for both types of coils. We have shown that the SNR of control coil is surpassed by the printed coils at phantom offsets of 18 mm using PEEK as dielectric and 28 mm using printed dielectric, when the printed coils are kept in close contact with the phantom. In our experiments, all coils could show additional losses from the cables used to connect to them when there is impedance mismatch, since we do not place preamplifiers directly on the coils. The calculated SNR considering the use of preamplifiers is shown as thin dotted lines in Fig. 3c (calculated with Supplementary Equations 3 and 4)^34^. When taking these losses into account, we find that printed coils based on PEEK show higher SNR when compared with control coils placed at 21 mm from the phantom. In addition, we show that SNR performance is minimally changed by the flexibility of the coils. The normalized SNR profiles around a curved and flat phantom of the same volume and composition are compared in Fig. 3d. The printed coils were wrapped around the phantom with a 22-mm radius of curvature. When placed in close proximity to the sample, coils are heavily loaded; therefore, changes in tuning due to the different geometry have a negligible effect on image SNR.

### The fabrication process is scalable to larger area coverage

**Figure 4:**
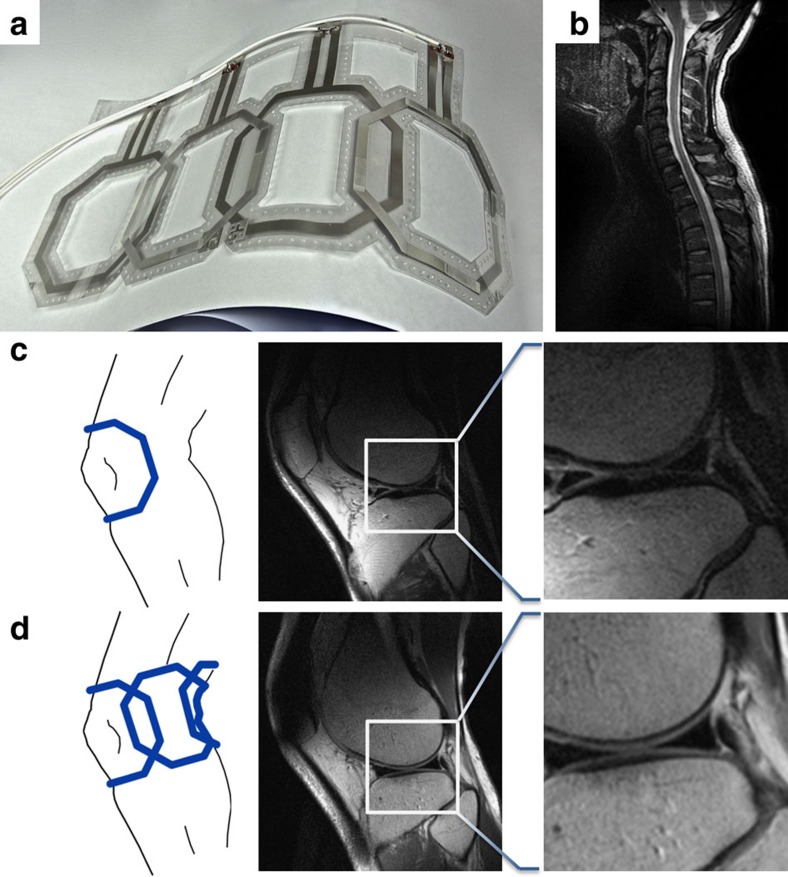
*In vivo* imaging with flexible coil array at 3 T. (**a**) Proof of concept, prototype of printed flexible four-channel receive array. (**b**) Sagittal cervical spine MRI image showing excellent penetration due to the conformity of the array. (**c**) Single-element MRI image of a knee. (**d**) Scan showing the expected improved penetration using a four-channel array wrapped around the leg of a volunteer. Highlighted areas show region of interest with higher SNR from increased field of view from array.

The focus of this work is on the design, fabrication and performance analysis of single surface coils. However, most clinical coils today come in arrays. We therefore believe it is important to demonstrate that our printing process is scalable to printing coil arrays. We therefore developed a proof of concept; a simple four-channel receive array to demonstrate array capacity of the process. The array was designed for 3 T and composed of four overlapped coil elements shown in Fig. 4a. This array was used as a proof of concept to image the cervical spine and knee, areas of the body where curvature can be a limiting factor. The cervical spine image is shown in Fig. 4b. The improved coverage and sensitivity of flexible coil arrays are illustrated by comparing knee images in Fig. 4c, taken with a single element, with Fig. 4d, taken with the array. Even though our prototype array did not focus on optimizing array geometry, coupling or fit, it produced high-quality images. Utilizing printing as a technique, other arrays with more elements can be built that include strain relief cuts, pre-curved substrates, more conformable materials or more advanced topography to better address even the most complex areas of the body—however, this advanced approach is beyond the scope of this preliminary work.

## Discussion

Here, we present the first fully functional, printed and flexible MRI coils, and array for 1.5-and 3-T clinical scanners. Our unique designs achieved a remarkable 80–93% of the control coils SNR depending on materials and construction. While current custom-built conventional coils have less intrinsic loss compared with current printed materials, it is impractical for custom traditional arrays to be built for each patient. The ease of adjustability in the printing process lends itself well to new geometries and materials, as was shown with our coil using the substrate as the dielectric. While printed coils with printed dielectric capacitors exhibit lower SNR in a one-to-one comparison with control coils, printed coils which use the PEEK substrate as the dielectric are less lossy. In fact, they near the performance of the control coils, in a typical sample-loaded, sample-noise dominated regime. At the same time, when the printed coils conform to patients and can be placed in close proximity to the body, they provide similar or better image quality than conventional ones that do not necessarily fit as well. The printed array integrated into a baby blanket is extremely lightweight and provides new opportunities for conformity and comfort with a mass manufacturing technique.

## Methods

### Coil fabrication

For coils with a printed dielectric, the first metal layer of the conductive coil was screen-printed, using an ASYS APM101 screen printer, onto a 75-μm-thick polyethylene terephthalate film using a silver micro-flake ink, with flake size of 7 μm, purchased from Creative Materials (118-19A/B). The metal layer was annealed at 125 °C for 15 min before the deposition of the dielectric material. Two types of dielectric inks were used when printing the tuning and matching capacitors, a ultraviolet-curable resin (Creative Materials 116-20) and a BaTiO_3_ ink (Conductive Compounds BT-101). A 60-μm-thick layer of ultraviolet-curable resin was used for 3-T coils and cured with a mercury arc lamp, with 24 W cm^−2^ power flux for 3 s. Coils designed for 1.5-T scanners required higher dielectric constant. For these, 30-μm-thick layer of BaTiO_3_ ink was used as the dielectric layer of the capacitors. After deposition, the BaTiO_3_ ink was annealed on a hot plate at 125 °C for 15 min. The top electrode of the capacitors was formed with a 30-μm-thick layer of silver micro-flake ink. The finished coil was further annealed at 125 °C for 15 min on a hot plate.

For coils with the substrate as the dielectric, the two metal layers on opposite sides of a 75-μm-thick PEEK film were printed using a silver micro-flake ink, purchased from Dupont (5064H). These layers were annealed at 140 °C for 15 min.

The control coils were fabricated using a 70-μm-thick etched copper on 75-μm-thick Pyralux AP low-loss substrate. Advanced Technical Ceramics 100B low-loss porcelain-based capacitors were soldered onto the copper traces to form tuned coils.

### Coil and component electrical characterization

Printed test capacitors were 5 mm wide and had an overlap ranging from 1 to 30 mm in length. Capacitors were mounted with plastic clamps on a copper PCB (printed circuit board) test fixture over a 30 × 30 mm^2^ opening in the board. For each measurement, the experimental fixture with capacitors was tested and calibrated on an Agilent E5061B ENA network analyzer with open, short and 50 Ω loads on an identically shaped calibration board. All coils were tuned with the phantom used to image via an S11 measurement on an Agilent E5061B ENA network analyzer. The printed tuning capacitors set the correct resonant frequency while the matching capacitors set the impedance. The capacitance values were varied by using different dielectric inks or changing the size of the metal electrodes. The optimization process was repeated until the coils resonated at the Larmor frequency and displayed 50 Ω impedance 31. Coil Q, was measured using an Agilent E5061B ENA network analyzer with two broadband magnetic field probes separated by 30 cm and facing each other to minimize the |S21| noise floor to approximately−90 dB (ref. 33). During all measurements, care was taken to ensure coils and test apparatus were at least 50 cm away from conductive material to prevent artificial loading of the coil. To measure loaded Q, coils were taped to a cubic phantom 7 litre in volume, filled with solution of 3.356 g l^−1^ NiCl_2_*6H_2_O and 2.4 g l^−1^ NaCl for conductivity of 0.68 Sm^−1^ at 123–127 MHz. Unloaded Q was measured using the magnitude of the S21 response with 1601 points averaged 16 times, centred at the Larmor frequency with the network analyzer set to a frequency span of 25 MHz, while loaded Q was measured with a span of 100 MHz.

### MRI Imaging

Single-channel cervical spine images were acquired with a turbo spin echo sequence on a 3-T Siemens scanner with an echo time (TE) of 112 ms, repetition time (TR) of 3500, ms and flip angle (FA) 90°. The field of view was 200 × 200 mm^2^ with resolution of 436 lines in phase encodes and readout directions with slice thickness of 4 mm. To compensate for imaging intensity variation due to the coil sensitivity, the image was normalized with respect to a uniform body coil image. SNR measurements were performed on the scanner by placing the coils on the same 7 litre phantom used to measure loaded Q. Coil bending tests were performed on different 7 litre phantoms containing a solution of 3 g l^−1^ of CuSO_4_ and 3 g l^−1^ NaCl. To interface with the scanner, all coils were clamped into a test fixture that had a PIN (p-type/intrinsic/n-type) diode to deactivate the coil during the transmit phase of each scan. This fixture was connected via half-wavelength long coaxial cables to an interface box made by Stark Contrast (Erlangen Germany), which housed preamplifiers connected to the scanner. The half-wavelength coaxial cable contained a cable-trap circuit tuned to the Larmor frequency. Image reconstruction was unchanged from that used in conventional coils.

The scans used to measure SNR were two-dimensional gradient echo sequences on 3-T Siemens and 1.5-T General Electric scanners with a TE of 10 ms, TR of 438 ms and FA of 25°. Field of view was 200 × 200 mm^2^ with resolution of 256 phase encodes and readouts. Slice thickness was 5 mm. The same prescan settings were used for all experiments, reducing variations due to differing magnet shimming, analogue gain and digital gain. Images of phantoms had SNR measured by dividing signal (that is, pixel in phantom), by an estimate of the noise sd. The noise was estimated from an image area with no signal containing at least 2,800 points at least 5 pixels from the edge of the image to avoid effects from the scanner’s low-pass filter. The noise only area did not contain any ringing or streaking artefacts in the phase encode direction. To maintain a uniform offset for the experiments, different thicknesses of polycarbonate sheet were inserted between the phantom and each coil.

### Fabrication and characterization

The four-channel array was fabricated by printing neighbouring coils on alternating sides of the substrate. The leads of each coil, along with a PIN diode, formed the dynamic disable circuit which detunes the coils during transmit. The PIN diode was attached using copper rivets pressed to the silver ink traces to form the electrical contact. The amount of coil overlap in the array was determined using two single coils and connecting each to one port of a network analyzer. Coils were overlapped until |S21| between the coils was minimized. The array was connected to low input impedance preamplifiers to take advantage of preamplifier decoupling, to reduce the amount of cross-channel coupling. Anatomy images of the spine taken with the printed array used a T2-weighted spin echo sequence: TE 114.8 ms, TR 3,500 ms, FA 90° and two averages on a 3-T General Electric scanner. The scan sequence for imaging a volunteer’s knee with a printed single-channel coil and with the four-channel array was a T2-weighted turbo spin echo with sequence parameters of TE of 39 ms, TR 3,000 ms, FA 150° and 1 average.

All experimental procedures were approved by the local ethical committee, Committee for protection of human subjects, University of California at Berkeley.

## Additional information

**How to cite this article**: Corea, J. R. *et al*. Screen-printed flexible magnetic resonance imaging receive coils. *Nat. Commun.* 7:10839 doi: 10.1038/ncomms10839 (2016).

## Supplementary Material

Supplementary Information Supplementary Figure 1, Supplementary Tables 1-6 and Supplementary Note 1
