# Supplementary material for: Screen-printed flexible MRI receive coils
Source: Nat Commun. 2016 Mar 10;7:10839. doi: 10.1038/ncomms10839 (PMC5553354; doi:10.1038/ncomms10839)
Supplement: Supplementary Information — Supplementary Figure 1, Supplementary Tables 1-6 and Supplementary Note 1 [file ncomms10839-s1.pdf]

Supplementary Figure 1: Sample Noise and Coil Noise regimes of operation for printed coils with coil radius and frequency.

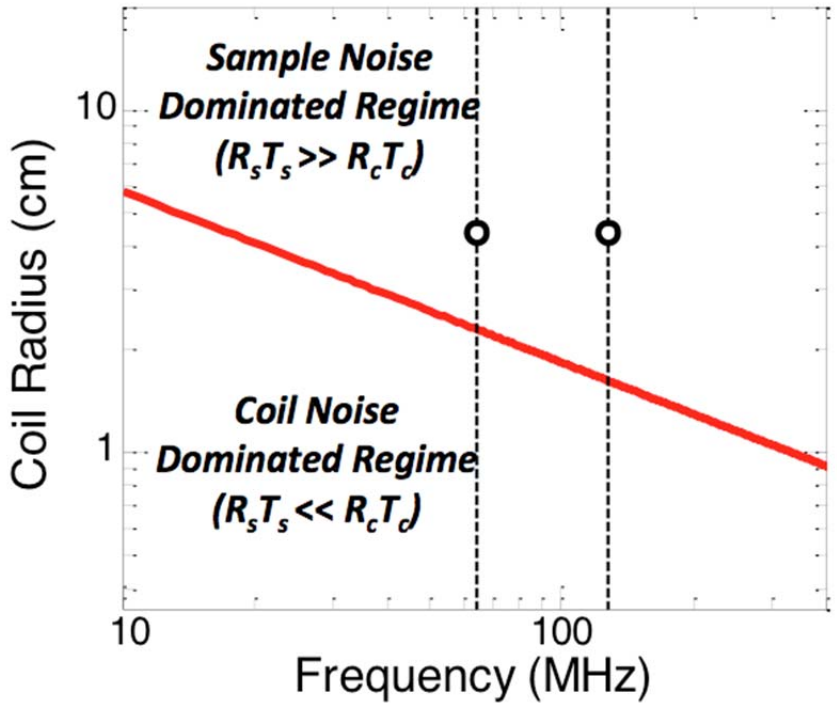

Dotted lines show frequencies of operation at 1.5 and 3T with circles showing the radius of our coils. The figure shows that our coils are in the sample noise dominant regime as they are above the red line in which sample noise and coil noise are equal.

Supplementary Table 1. Loaded, unloaded, predicted, and measured SNR for coils created for 3 T system. Several regions within the phantom are used to calculate SNR.

Coil Type	$Q_{unloaded}$	Q_{loaded}	Predicted SNR vs. Control	Measured SNR vs. Control			
				All	3-5 cm	5-7 cm	>7cm
Control (non-printed)	395	11.4	100 %	100 %	100 %	100 %	100 %
Printed Conductor	76	9.8	93 %	96±3 %	96±3 %	94±4 %	93±5 %
Substrate Capacitor	73	9.5	91 %	93±3%	93±3%	94±4 %	95±5 %
Printed Capacitor	22.3	7.6	82 %	86±3 %	86±3 %	83±4 %	82±5 %
All Printed	17.9	6.7	77 %	79±3 %	80±3%	78±4 %	78±5 %

Supplementary Table 2. Loaded, unloaded, and predicted SNR for coils created for 1.5 T system. Several regions within the phantom are used to calculate SNR.

Coil Type	$Q_{unloaded}$	Q_{loaded}	Predicted SNR vs. Control	Measured SNR vs. Control			
				All	3-5 cm	5-7 cm	>7cm
Control (non-printed)	367	28.9	100 %	100%	100 %	100 %	100 %
All Printed	23	13.2	67 %	69±3 %	68±3 %	70±4 %	70±5 %

Supplementary Table 3. SNR data for coil offset testing on 3T system with signal from entire phantom. Colors highlight approximate location where SNR is equal for different offsets.

	Measured SNR in entire phantom vs. Offset (% of control)								
Offset	0 mm	6 mm	12 mm	18 mm	24 mm	30 mm	36 mm	42 mm	48 mm
Control	100	99±3	97±3	92±3	85±3	77±3	70±3	63±3	57±3
PEEK Cap	92±3	91±3	86±3	79±3	71±3	63±3	57±3	50±3	44±3
All Printed	79±3	76±3	69±3	62±3	54±3	48±3	41±3	36±3	32±3

Supplementary Table 4. SNR data for coil offset testing on 3T system with signal from 3-5 cm into phantom. Colors highlight approximate location where SNR is equal for different offsets.

	Measured SNR 3cm-5cm into phantom vs. Offset (% of Control)								
Offset	0 mm	6 mm	12 mm	18 mm	24 mm	30 mm	36 mm	42 mm	48 mm
Control	100	96±3	92±3	86±3	79±3	71±3	64±3	57±3	52±3
PEEK Cap	93±3	90±3	83±3	74±3	65±3	57±3	51±3	44±3	38±3
All Printed	78±3	72±3	64±3	57±3	49±3	43±3	37±3	32±3	28±3

Supplementary Table 5. SNR data for coil offset testing on 3T system with signal from 5-7 cm into phantom. Colors highlight approximate location where SNR is equal for different offsets.

	Measured SNR 5cm-7cm into phantom vs. Offset (% of Control)								
Offset	0 mm	6 mm	12 mm	18 mm	24 mm	30 mm	36 mm	42 mm	48 mm
Control	100	96±4	93±4	88±4	81±4	75±4	69±4	62±4	57±4
PEEK Cap	95±4	91±4	84±4	77±4	69±4	61±4	55±4	49±4	43±4
All Printed	77±4	72±4	65±4	58±4	51±4	46±4	40±4	36±4	32±4

Supplementary Table 6. SNR data for coil offset testing on 3T system with signal from 7 cm and deeper in phantom. Colors highlight approximate location where SNR is equal for different offsets.

	Measured SNR >7 cm into phantom vs. Offset (% of Control)								
Offset	0 mm	6 mm	12 mm	18 mm	24 mm	30 mm	36 mm	42 mm	48 mm
Control	100	97±5	95±5	92±5	87±5	81±5	76±5	70±5	65±5
PEEK Cap	96±5	93±5	88±5	82±5	75±5	69±5	64±5	59±5	54±5
All Printed	77±5	72±5	67±5	61±5	55±5	50±5	45±5	41±5	38±5

Supplementary Note 1

Supplementary Equations

(1)
$$R_C = \sqrt{0.5\rho_c\mu_0\omega}\left(\xi\frac{a}{r}\right)$$

(2)
$$R_s \approx \frac{2}{3\pi}\sigma\mu_0^2\omega^2a^3 arc\tan\left[\frac{\pi a}{8d}\right]$$

(3)
$$|\rho| = \frac{VSWR - 1}{VSWR + 1}$$

(4)
$$Total\ Mismatched\ Line\ Loss = 10\log\left[\frac{a^2 - |\rho^2|}{a\,(1 - |\rho^2|)}\right]$$
